# Effects of fatigue on microstructure and mechanical properties of bone organic matrix under compression

**DOI:** 10.1007/s13246-013-0185-1

**Published:** 2013-02-08

**Authors:** Hanna Trębacz, Artur Zdunek, Justyna Cybulska, Piotr Pieczywek

**Affiliations:** 1 Department of Biophysics, Medical University of Lublin, al. Racławickie 1, 20-059, Lublin, Poland; 2 Institute of Agrophysics Polish Academy of Sciences, ul. Doświadczalna 4, 20-290 Lublin, Poland

**Keywords:** Acoustic emission, Anisotropy, Bovine cortical bone, Fatigue, Image analysis

## Abstract

The aim of the study was to investigate whether a fatigue induced weakening of cortical bone was revealed in microstructure and mechanical competence of demineralized bone matrix. Two types of cortical bone samples (plexiform and Haversian) were use. Bone slabs from the midshaft of bovine femora were subjected to cyclical bending. Fatigued and adjacent control samples were cut into cubes and demineralized in ethylenediaminetetraacetic acid. Demineralized samples were either subjected to microscopic quantitative image analysis, or compressed to failure (in longitudinal or transverse direction) with a simultaneous analysis of acoustic emission (AE). In fatigued samples porosity of organic matrix and average area of pores have risen, along with a change in the pores shape. The effect of fatigue depended on the type of the bone, being more pronounced in the plexiform than in Haversian tissue. Demineralized bone matrix was anisotropic under compressive loads in both types of cortical structure. The main result of fatigue pretreatment on mechanical parameters was a significant decrease of ultimate strain in the transverse direction in plexiform samples. The decrease of strain in this group was accompanied by a considerable increase of the fraction of large pores and a significant change in AE energy.

## Introduction

Bone tissue can be considered as a hierarchical composite material consisting mainly of type I collagen, crystals of hydroxyapatite and water. To fulfill diverse biological and mechanical functions in bone that basic material is arranged into complex structures at a wide range of length scales. The collagen fibrils (100–200 nm in diameter), with thin elongated mineral platelets of hydroxyapatite inside and between them are arranged into fibril arrays or lamellae (3–7 μm wide) forming the basic structure of bone tissue [1]. This study focuses on bovine cortical bone, the microstructure of which has been classified into two main types: plexiform and Haversian [2, 3]. The plexiform microstructure is made of layers of brick shape vascular plexuses sandwiched within the laminar tissue. Plexiform layers have a thickness of about 100–200 μm. Haversian microstructure is made of osteons, which are 150–300 mm wide cylindrical structures made of 3–8 lamellae surrounding the Haversian canal. Their axes are principally aligned parallel to the long axis of the bone [1].

From the mechanical point of view, mineralized bone tissue is a brittle material deriving its resistance against fracture by absorbing energy in a form of microcracks that provide an appropriate ductility of the material and delay the propagation of fracture [4, 5]. So, a broad area of interest in bone research is the role of microdamage accumulation in declining mechanical properties of bone due to aging, overloading or impaired bone metabolism [6–8]. A number of studies revealed that mechanisms of bone failure are dependent on the effectiveness of energy dissipation within the tissue [9–12]. It is expected that studies on interaction of fatigue damage with bone microstructural features and composition will provide better understanding of bone fracture.

Even though the load bearing capacity in bone is supplied essentially by mineral phase, which has been known for more than 30 years [13–16], an increasing number of studies underlines a crucial role for collagen quality and microstructure for energy absorbed in bone during deformation and for fracture toughness [6, 17–21]. Any change of deformability of bone matrix collagen can reduce ductility of bone material and impair its toughening behavior, resulting in an increasing propensity of bone to undergo a brittle failure.

A few previous attempts have been made to characterize mechanical behavior of a collagenous matrix obtained from demineralized bone using different experimental approaches [7, 22–27]. The studies have demonstrated a large range of values for measured properties depending on the structural level, type of bone and a type and procedure of loading. However, to the best of our knowledge there was no study of the impact of fatigue loads on structural and mechanical properties of organic matrix of cortical bone.

The aim of the present study was to investigate whether a fatigue induced weakening of cortical bone was revealed in microstructure and mechanical competence of demineralized bone matrix. The other question addressed in this study was whether the response of collagenous matrix of the fatigued bone to compression was directionally dependent.

## Methods

### Sample preparation

**Fig. 1:**
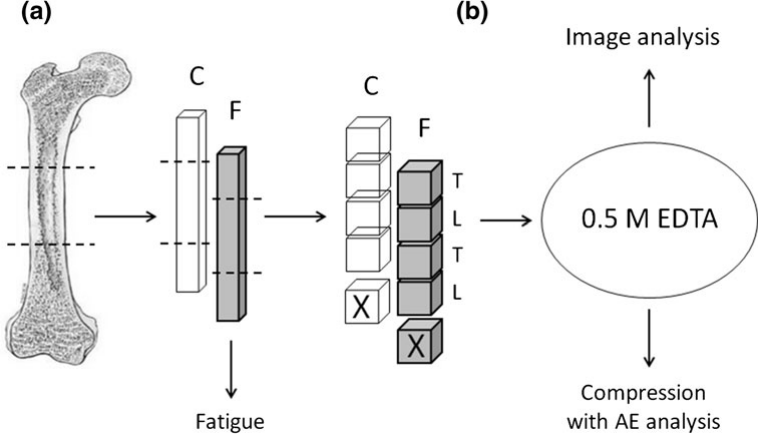
Scheme of samples preparation: **a**. Bone slabs for fatigue experiment (F) and adjacent controls (C), **b**. further treatment of samples-demineralization in EDTA and then: image analysis or unconfined compression (F,C,L,T), or ashing (X); L, T–samples subjected to compression in longitudinal (L) or transverse (T) direction

Samples were prepared from a pair of bovine femora of 18 months old cattle. One cross-sectional segment (55 mm in length) was cut from the mid diaphysis of each femur. Each segment was dissected along the longitudinal axis into twelve bone strips—three pairs from antero-medial (A-M) and three pairs from postero-lateral (P-L) aspect of cortex. The central part of each strip was machined into a rectangular specimen 50 mm × 5.0 mm × 5.0 mm (Fig. 1a). Special attention was paid to obtain the surfaces of each sample as parallel as possible. Finally, there were 24 bone slabs: six pairs from A-M and six pairs from P-L part of femur cross-section. It was stated previously, that two histologically different structures of cortical bone can be found in bovine femur [2, 3, 28]. Haversian microstructure was mainly found in P-L regions and plexiform microstructure in A-M regions of the bone. The longest dimension of samples was along the main axis of the femur. Two other dimensions were along circumferential and radial directions, however no attention was paid to distinguish these two directions during testing procedures. The samples were stored in 0.9 % NaCl solution in 4 °C prior to fatigue.

### Fatigue

One from each pair of bone slabs, (group F: 6 A-M and 6 P-L samples), was subjected to 300 cycles of three-point bending to constant deflection 0.9 mm at 100 mm/min (group F) using a Lloyd LRX machine with a 2500 N load cell (Lloyd Instruments Ltd., UK). The span of supports was 32 mm. During the loading cycle (4–5 min for one sample) the hydration of specimens was maintained. The other slab from each pair was left as control (group C).

A preliminary experiment was carried out to determine a combination of number of cycles and deflection to obtain a significant fatigue (more than 5 % of force decrease observed in three subsequent samples) without destroying of the samples. However, in the main experiment two samples failed before the cycles were completed: one at 220th cycle and the other at 280th one.

### Demineralization

After fatigue, the bone samples, both F and C, were cut with a diamond blade into 5.0 mm cubes. Four cubes were obtained from the middle part of each 50 mm long sample, which corresponded to the region subjected to bending in fatigued samples (Fig. 1b). In each cube the direction parallel to the long axis of bone was marked as longitudinal (L). As the two bone slabs that had failed during the fatigue cycle had fractured in the middle, it was possible to cut the cubes also from these samples and to include them in further procedures.

All F (*n* = 48) and C (*n* = 48) bone cubes together with samples X cut from the end parts of bone slabs were demineralized in a neutral solution of 0.5 M EDTA (ethylenediaminetetraacetic acid) for six weeks at room temperature. The solution was stirred twice a day and changed twice a week. Completeness of demineralization was checked by ashing samples X in a muffle furnace at 620^º^ C for 10 h [29].

### Microscopic observation and image analysis

Two sets of demineralized bone cubes were used for image analysis: one set was from the center of A-M and the other-from the center of P-L part of cortex. Each set consisted of eight cubes: four from a fatigued bone and four from adjacent controls. Slices 100 μm thick were cut from each cube with Vibratome LEICA VT 1000S. Slices were stained according to Mayer’s hematoxylin & eosin procedure. Images were captured with use of a confocal scanning laser microscope (OLYMPUS FluoView300, Olympus Corp., Japan). Images were acquired at 4 times magnification using UPlanSApo 4x/0.16 objective giving a resolution of 2.971 μm^2^ per pixel. The image size was equal to 2048 × 2048 pixels, which corresponded to field of observation of 3.53 mm × 3.53 mm. For each sample 3 or 4 microscopic slices were analyzed.

Image processing was aimed at separation and quantification of all non-collagenous spaces within the sample, regardless of their origin. The separated spaces were called “objects”. It was supposed that the objects separated from the microscopic images comprise all pores, voids, fissures and fracture spaces in the sample.

Grey-scale images (0–255 scale) were processed from microscopic images using a protocol developed in Matlab R2010a (MathWorks, U.S.A.) (Fig. 2). Median filtering 5-by-5 pixels was used to reduce “salt and pepper” noise and then a procedure of equalization of image brightness was applied. A strong Gaussian blur filter used in this procedure removed relative small details from the image. The value of each pixel from the original image was divided by the value of the corresponding pixel from the blurred image. To separate wanted structures from the background of the image the segmentation by fixed gray level threshold value of 50 was done. The boundary objects, visible only in part were not taken into account in further analysis (Fig. 2 b).

**Fig. 2:**
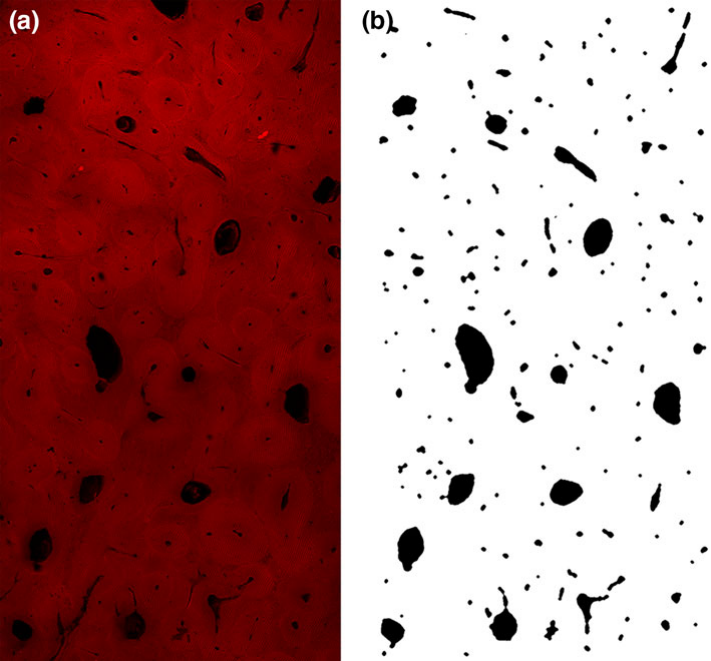
An example of a microscopic image of a decalcified Haversian bone sample (P-L) (**a**), and a numerically processed image of that sample (**b**); Images were captured using OLYMPUS FluoView300 (Olympus Corp., Japan) with resolution of 2.971 μm^2^ per pixel

**Fig. 3:**
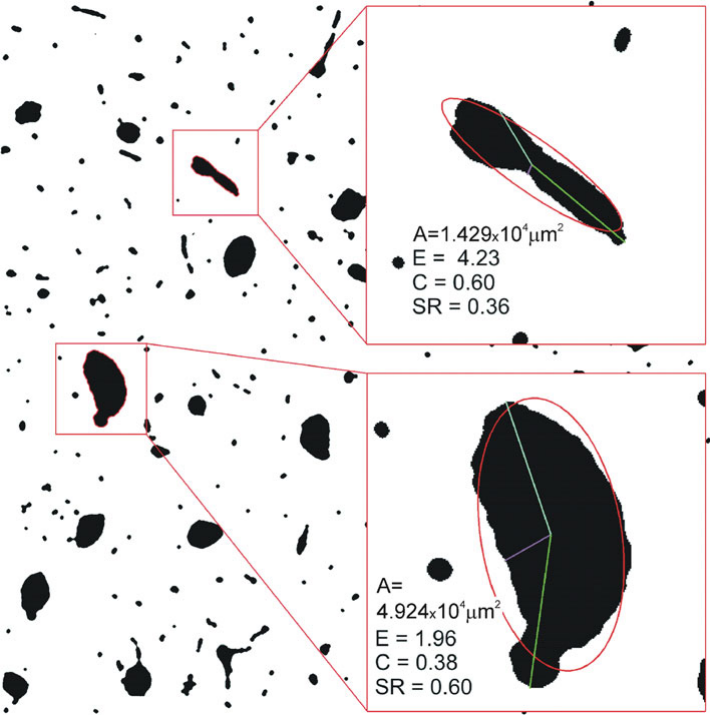
An example description of two different objects from microscopic image of a decalcified bone sample (the sample shown in Fig. 2) in terms of applied image analysis; *A*-the object’s area, *E*-elongation calculated from the best fitted ellipse, *C*-circularity and *SR*-shape roughness calculated from the maximum, minimum and median radius of the region

Four parameters were calculated for each object (Fig. 3). Area (A) was calculated from the number of pixels within the object, considering that one pixel corresponded to 2.971 μm^2^. Elongation (E) was defined as a ratio of semi-major to semi-minor axis of the best fitted ellipse that had the same second moment of inertia as the analyzed region [30]. The deviation from the circular shape was expressed by a circularity coefficient (C) (according to Documentation of Image Processing Toolbox for use with Matlab):

1 $$ C = 1 - \frac{4 \cdot \pi \cdot A}{{P^{2} }} $$

where *A-*area and *P*-perimeter.

For a circle, C was equal to 0.

Shape roughness (SR) was given by the equation [31]:

2 $$ SR = \root{3} \of {{\frac{{{\text{med}}\left( {d\left( {x,y} \right)} \right) \cdot \hbox{min} \left( {d\left( {x,y} \right)} \right)}}{{\left[ {\hbox{max} \left( {d\left( {x,y} \right)} \right)} \right]^{2} }}}} $$

where *d(x,y)*-the distance of the point from object’s perimeter to the centroid of the object.

The less regular was the shape, the lower was SR.

Average values of the geometric parameters for objects from all slices in each group of samples (A-M/C, A-M/F, P-L/C and P-L/F) were calculated, however distribution of parameters’ values was examined on the basis of histogram analysis. Additionally, total pore surface area was estimated for each bone slice as sum of area of objects in the field of observation divided by the total area observed.

### Compression

Demineralized bone cubes, except for 16 samples used for image analysis, were subjected to unconfined compression (40 fatigue and 40 control samples). In both groups half of samples were compressed parallel to the long axis (L) and the other half in a perpendicular direction (T) (Fig. 1b). Samples from A-M and from P-L parts of cortex were analyzed separately as different groups. So, there were 8 groups of samples, 10 samples in each.

Samples were compressed at a constant strain rate 0.033 s^−1^ up to 3 mm displacement of the cross-head of the Lloyd LRX testing machine. Recording of the deformation-load curve was triggered at the force passing 0.1 N, where the sample deformation in the vertical direction was the same as the cross-head displacement. The tests were done without any preconditioning with the samples fully wet.

### Acoustic emission analysis

The acoustic emission (AE) signal was recorded during sample compression. AE was measured with an AE head connected with the compression plate of the testing machine as previously described [27]. A 4381 V acoustic sensor (Bruel & Kjear, Denmark) with a maximum sensitivity in the range 1–16 kHz glued inside the head was connected to the signal amplifier (EA System S.C., Poland). After filtering, the signal was converted using A/D board PCI 9112 (Adlink Technology Inc., Taiwan). The sampling rate was 44,000 samples/second at a resolution of 16 bits/± 1.25 V. An analogue signal of force delivered from the testing machine was recorded using the second channel of the A/D board in order to trigger both AE and force measurements simultaneously.

AE events and AE energy were used to characterize the material [27]. AE events number and AE energy were counted every 0.1 s during the entire test. In the transversely loaded (T) samples, cumulative values of both AE descriptors were calculated from the start of compression to 95 % of maximum load to avoid signals arising from the final fracture. In the longitudinally loaded (L) samples AE was recorded from the start to 50 % strain, which was a consequence of qualitatively different results of compression for T and L.

### Statistical analysis

Effectiveness of the fatigue procedure was estimated using paired *t* test. Results of image analysis and compression of demineralized bone samples were analyzed in terms of two-way ANOVA, where histological type (A-M vs P-L) and pre-treatment (fatigue vs control) were considered as factors. ANOVA was followed by Tuckey’s post hoc analysis. Statistical tests were performed using Statistica 9 (StatSoft Inc., USA). The 5 % level of significance was applied for all tests.

## Results

### Cyclic bending

For each of twelve fatigued bone slabs the secant modulus obtained from the last complete cycle of bending (M_last_) was compared with the initial modulus (M_first_) in order to check the assumption that the bending procedure affected mechanical properties of the samples. During 300 cycles the modulus decreased by 5.7 %, in average, from M_first_ = 142.3 MPa (S.D. = 19.2 MPa) to M_last_ = 134.2 MPa (S.D. = 18.8 MPa). There was no statistically significant difference (*t* test) between A-M and P-L slabs as concerns M_last_, M_first_ and the change of modulus during fatigue. The difference between M_last_ and M_first_ for all fatigued bone slabs was significant in terms of paired *t* test (*p* = 0.0008). Accounting for this difference, a kind of bending induced destruction of bone tissue was inferred.

### Demineralization

After six weeks of treatment in EDTA test samples X (see Fig. 5) were annealed with no residuum, so demineralization of all bone samples was assumed to be complete. Demineralization resulted in a change in dimensions of initial bone cubes, so as after the process the samples were not regular cubes but parallelepipeds, 4.73 (S.D. = 0.14) mm × 4.89 (0.09) mm × 4.93 (0.06) mm, in average, in respect to 5.01(0.06) mm × 5.00(0.04) mm × 5.01(0.04) mm in the intact samples. The differences between dimensions of samples before and after demineralization were significant in terms of *t* test (*p* < 0.01). However, no statistically significant difference of average dimensions was seen between F and C, or between A-M and P-L samples after demineralization in terms of two-way ANOVA (*p* = 0.216).

### Image analysis

**Fig. 4:**
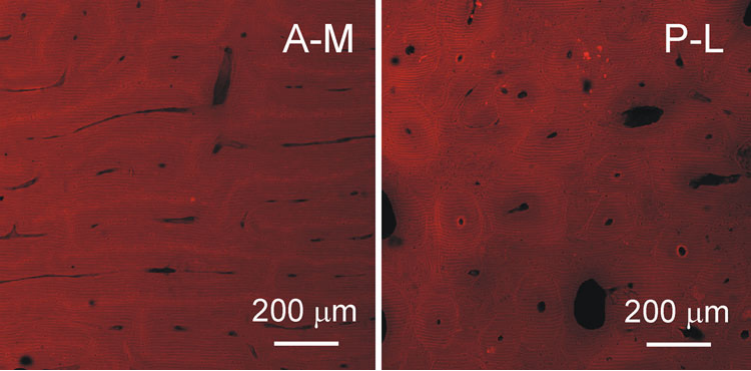
Microscopic images of demineralized control bone samples from two histologically different parts of bovine femur’s cross-section: antero-medial (A-M)–plexiform, and postero-lateral (P-L)–Haversian

The microscopic image analysis of control samples confirmed that two histologically different cortical structures were used in the study (Fig. 4). A-M samples, as reported previously for plexiform bone, were made of brick like structures arranged into regular lamellae [2, 3, 28]. P-L samples revealed a Haversian microstructure.

Total pore fraction was significantly different between the groups (Table 1). Porosity of samples was influenced significantly by both factors: histological type (A-M vs P-L) and fatigue (F vs C). However, there was no significant interaction between these factors. Post-hoc analysis revealed that porosity of the Haversian samples was higher than of the plexiform ones, both in the fatigued and control groups, and that fatigue resulted in a significant increase of porosity in Haversian bone.

**Table 1:** Total pore surface area obtained from microscopic images of demineralized bone samples from fatigued (F) and control (C) bone in two histologically different parts of femoral shaft (A-M–plexiform, and P-L–Haversian); mean values and S.D. (in parenthesis) are given, *n–*number of microscopic slices analyzed in the group; In the lower panel *p* values from two-way ANOVA are shown presenting significance of differences between groups in the full model, for both factors (histological type and fatigue) and interaction between factors

A-M		P-L	
F; *n* = 12	C; *n* = 12	F; *n* = 16	C; *n* = 16
3.01 % (1.17 %)^###F,C^	2.34 % (0.50 %)^###F,C^	7.45 % (2.01 %) **	5.80 % (1.61 %)

Full model	A-M vs P-L	F vs C	Interaction
<0.000	<0.000	<0.000	0.232

Tukey’s post-test: *, **, ***–significantly different from the control in the same histological group (*p* < 0.05, <0.02 and <0.01, respectively); ^#^, ^##^, ^###^–significantly different from F,C in the other histological group (*p* < 0.05, <0.02 and <0.01, respectively)

Average values of geometric parameters referring to the area (A) and shape (E, C, SR) of pores (“objects” on the image) for each type of samples are presented in the Table 2. For all parameters the differences between the groups were extremely significant, both considering histological type and fatigue pre-treatment. Moreover, there was a significant interaction between both factors for all shape descriptors (E, C, SR) indicating that fatigue influenced the shape of pores in a way dependent on the histological type. In A-M samples (plexiform) fatigue resulted in less elongated, more circular objects with more regular perimeter, while in P-L samples (Haversian) elongation and shape roughness of objects increased. All these differences were significant in terms of post hoc analysis. In regard to average area of objects (A), the objects in P-L samples were significantly larger than in A-M. There was also a significant increase of A after fatigue: by 15 % in P-L and as much as 60 % in A-M samples.

**Table 2:** Results of image analysis for demineralized bone samples: control (C) and fatigued (F), from antero-medial (A-M) and postero-lateral part of femoral shaft; A–area of object, E–elongation, C–circularity, SR–shape roughness; mean values and S.D. (in parenthesis) are given; *n*–number of objects analyzed on microscopic slices; In the lower panel *p* values from two-way ANOVA are shown presenting significance of differences between groups in the full model, for both factors (histological type and fatigue) and interaction between factors

	A-M		P-L	
	F; *n* = 3125	C; *n* = 3880	F; *n* = 5228	C; *n* = 4695
A (μm^2^)	1423 (3295)***	888 (2600)	2822 (9026)**,^###F/C^	2445 (7888)^###F/C^
E	2.24 (1.25)***	3.39 (2.32)	2.21 (1.28)***,^###C^	2.08 (1.14)^###F/C^
C	0.311 (0.213)***	0.399 (0.214)	0.334 (0.224)***,^###F/C^	0.303 (0.215)^###C^
SR	0.631 (0.159)***	0.562 (0.149)	0.626 (0.162)***,^**###C**^	0.648 (0.161)^**###F/C**^

*p* values	Full model	A-M vs P-L	F vs C	Interaction
A	<0.000	<0.000	<0.000	0.461
E	<0.000	<0.000	<0.000	<0.000
C	<0.000	<0.000	<0.000	<0.000
SR	<0.000	<0.000	<0.000	<0.000

Tukey’s post-test: *, **, ***-significantly different from the control in the same histological group (*p* < 0.05, <0.02 and <0.01, respectively); ^#^, ^##^, ^###^-significantly different from F/C in the other histological group (*p* < 0.05, <0.02 and <0.01, respectively)

**Fig. 5:**
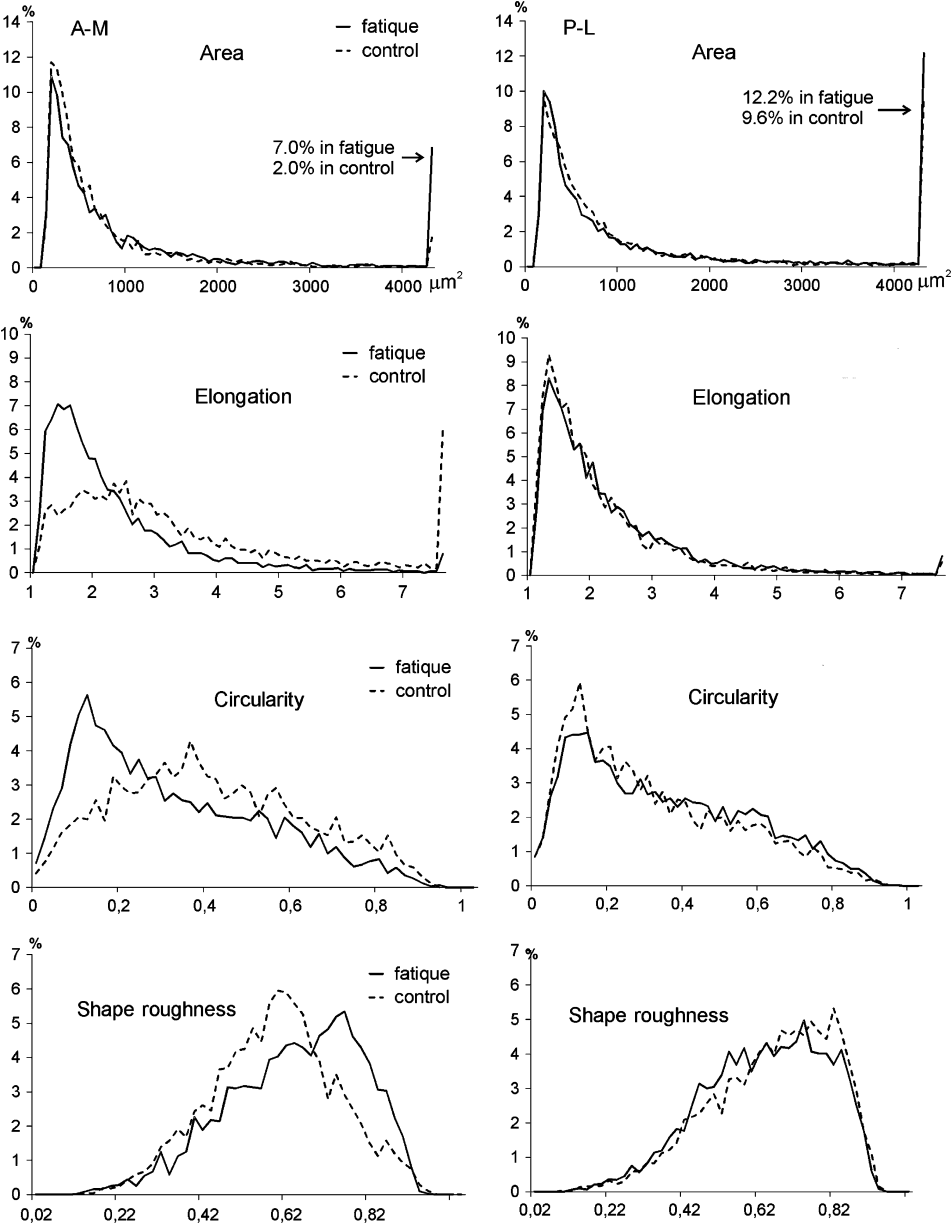
Histograms of geometric descriptors of pores (objects) on microscopic images of demineralized samples from fatigued (*solid line*) and control (*dotted line*) bone; *left* panels–A-M (plexiform) samples; *right* panels –P-L (haversian) samples; In each panel, only the envelope of the histogram is shown instead of *bars*. The intervals for the histograms were as follows, 60 μm^2^ for the surface area, 0.1 for the elongation, 0.02 for the circularity and 0.02 for the shape roughness

Geometry of the objects on the microscopic images of samples was also analyzed in the terms of distribution histograms showing frequency of observations for discrete intervals of values (Fig. 5). For clarity, instead of a system of narrow bars, only its envelope is shown in each panel. The main body of objects in control samples, 80.0 % in A-M and 67.9 %, were small, with the area less than 1000 μm^2^. Approximately 20 % of objects in both groups had the area between 200 and 300 μm^2^. There was also 9.6 % of objects larger than 4000 μm^2^ in P-L compared to 2 % in A-M. After fatigue, the contribution of the objects smaller than 1000 μm^2^ dropped to 70.6 % in A-M and the fraction of the largest objects (>4000 μm^2^) increased to 12.2 % in P-L and to 7.0 % in A-M. There was also a considerable shift of histograms for shape descriptors in A-M samples revealing a smaller number of extremely elongated objects (E > 7.5) and a general shift into lower elongations and a more regular perimeter. The distribution of shape descriptors in P-L was less influenced by fatigue, though a larger number of irregular objects with rough perimeter appeared.

### Compression tests with acoustic emission

**Fig. 6:**
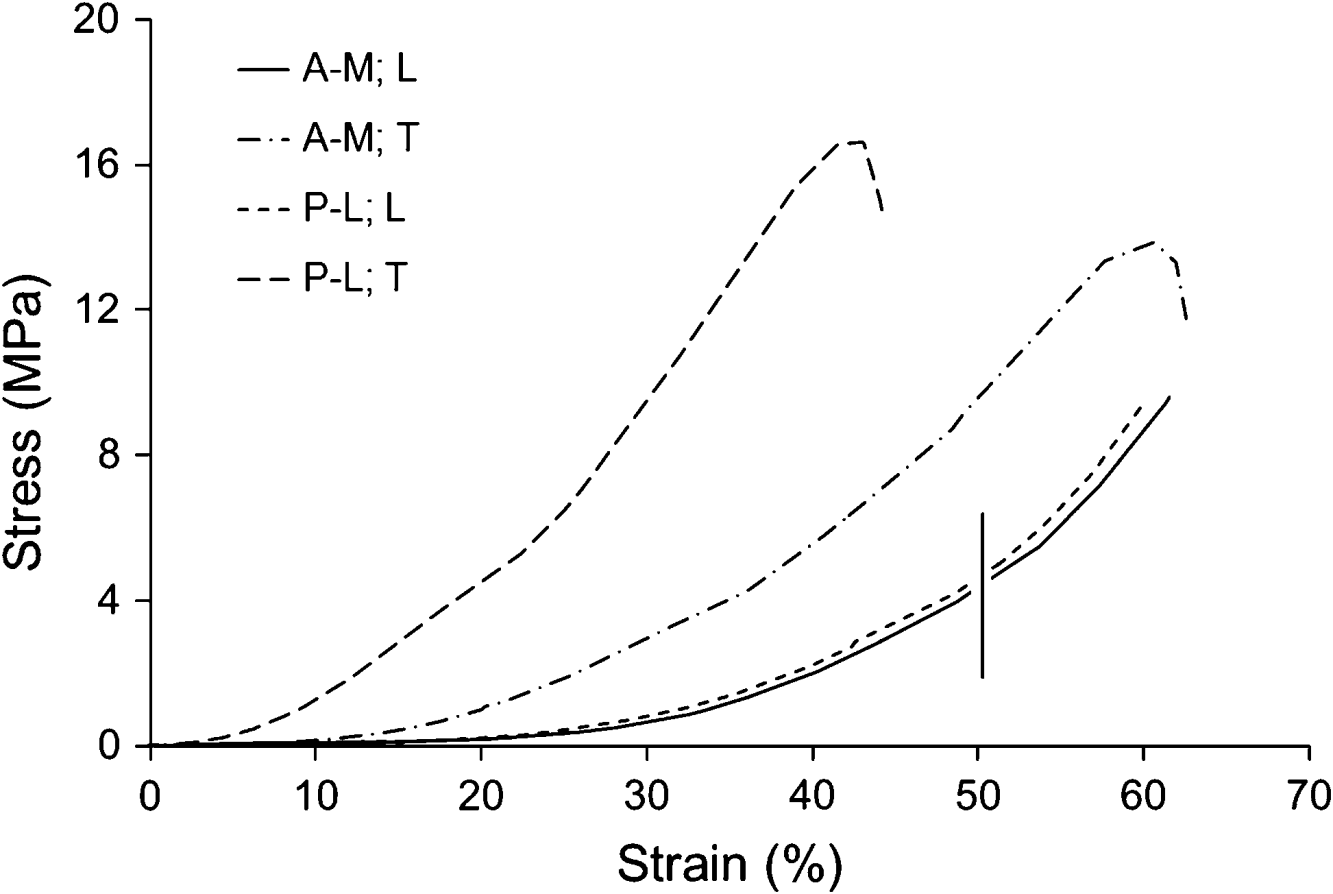
Examples of compression stress–strain curves for longitudinally (L) and transversely (T) loaded control samples of demineralized bovine bone from two histologically different groups (A-M–plexiform, and P-L–Haversian); L samples were analyzed to 50 % strain point

The mechanical behavior of demineralized bone samples was characterized on the basis of strain–stress relationship and in terms of work obtained from the area under the deformation–load curve. Bone matrix deformed nonlinearly for both directions of loading, exhibiting also highly anisotropic properties, both in control and fatigued samples. Figure 6 shows examples of stress–strain curves for L and T samples from two different control groups: A-M–plexiform, and P-L–Haversian. The shapes of other curves in each group were very similar to these presented in Fig. 6.

The deformation up to 3 mm was sufficient to cause failure of each of T samples, while none of L samples was ultimately fractured. Therefore, L samples were analyzed at 50 % strain, which was arbitrary, but close to ultimate strains obtained for L samples. There was no significant difference in stress and work at 50 % strain between L groups (Table 3). However, acoustic emission during compression in the longitudinal direction was significantly different between groups. Two-way Anova for L samples revealed an impact of fatigue on both AE events and energy, while histological type influenced the energy but not the number of AE events. In A-M samples from fatigued bone AE energy was significantly larger than from the control.

**Table 3:** Results of compressive test for demineralized bone samples loaded in longitudinal direction (L); values obtained for 50 % strain; mean value S.D. (in parenthesis) in groups are given, *n*–number of samples in the group

	A-M		P-L	
	F; *n* = 10	C; *n* = 10	F; *n* = 10	C; *n* = 10
Stress (MPa)	5.23 (1.70)	4.81 (0.79)	5.33 (1.43)	5.48 (1.59)
Work (mJ)	107 (28)	99 (16)	113 (33)	109 (26)
AE events (a.u.)	339 (54)	258 (52)^#F/C^	344 (82)	335 (78)
AE energy (a.u.)	16.69 (5.26)*	11.06 (3.50)^#F/C^	17.77 (6.04)	17.11 (6.21)

*p* values	Full model	A-M vs P-L	F vs C	Interaction
Stress	n.s.	–	–	–
Work	n.s.	–	–	–
AE events	0.0429	n.s.	0.0409	n.s.
AE energy	0.0172	0.0463	0.0402	n.s.

Tukey’s post-test: *, **, *** -significantly different from the control in the same histological group (*p* <  0.05, <0.02 and <0.01, respectively); ^#^, ^##^, ^###^- significantly different from F/C in the other histological group (*p* < 0.05, <0.02 and <0.01, respectively)

Mechanical properties of T samples were characterized in terms of ultimate stress, strain and work to failure (Table 4). Strain at failure was significantly different between groups with *p* = 0.0013. Strain was significantly influenced both by fatigue (*p* = 0.004) and by histological type (*p* = 0.0091). There was also a significant impact of both factors on energy of AE. However, an interaction between both factors was not significant. Post-hoc analysis between pairs of groups showed a significantly lower ultimate strain in fatigued plexiform samples as compares to controls, but energy of acoustic emission released during deformation in fatigued T samples was higher than in controls. There was also a significant difference between control plexiform and Haversian samples, both considering strain and AE energy.

**Table 4:** Results of compressive test for demineralized bone samples loaded in a transverse direction (T); mean value S.D. (in parenthesis) in groups are given; *n*–number of samples in the group

	A-M		P-L	
	F; *n* = 10	C; *n* = 10	F; *n* = 10	C; *n* = 10
Stress at failure (MPa)	14.55 (3.38)	15.0 (3.36)	16.39 (3.14)	16.48 (3.43)
Strain at failure (%)	48.6 (8.0)***	58.3 (7.2)^###F/C^	45.78 (5.92)	47.21 (6.19)
Work at failure (mJ)	249 (48)	290(58)	292 (40)	295 (65)
AE events (a.u.)	316 (44)	273 (57)	322 (73)	311 (63)
AE energy (a.u.)	19.00 (7.03)*	14.40 (4.40)^**###F/C**^	22.46 (6.89)	20.60 (5.27)

*p* values	Full model	A-M vs P-L	F vs C	Interaction
Stress	n.s.	–	–	–
Strain	0.0013	0.0091	0.0040	n.s
Work	n.s.	–	–	–
AE events	n.s.	–	–	–
AE energy	0.0213	0.0412	0.0319	n.s.

Tukey’s post-test: *, **, ***- significantly different from the control in the same histological group (*p* < 0.05, <0.02 and <0.01, respectively); ^#^, ^##^, ^###^- significantly different from F/C in the other histological group (*p* < 0.05, <0.02 and <0.01, respectively)

For both directions of loading (L and T) emission of acoustic energy occurred in the whole range of deformation. Nearly uniform emission of acoustic signals with low energy was accompanied by more or less regular occurrences with energy 5–15 times higher than the basic signal.

## Discussion

In the present study microstructure and mechanical competence of demineralized bone matrix from fatigued cortical bone were investigated. It should be underlined that the study was focused on the overall quantitative estimation of fatigue induced changes without investigation of their nature. However, two histologically different types of cortical tissue, plexiform and haversian, were distinguished. The microscopic analysis was aimed at quantification of all non-collagenous spaces in the demineralized bone: pores, voids, canals, fissures etc., regardless of their origin. It was supposed that any change in microstructure of organic matrix in cortical bone should be reflected in size and/or shape of these objects. In fatigued samples, a basic image of the structures was not changed being typical for plexiform bone in A-M and Haversian in P-L. However, quantitative and qualitative changes in matrix structure were noticed. Porosity of fatigued samples and average area of objects have risen, along with a change in the shape profile, especially in the plexiform bone. Considering, that fatigue loading produces microdamages which are small objects [4, 17, 21, 32] and that they tend to appear at edges of canals, voids and bone cells lacunae, which are typical stress concentrators in bone tissue [5, 6, 9, 10, 17, 32, 33], one can presume that in the present experiment fatigue resulted in an enlargement of existing pores rather than in creating new, separate objects. Moreover, during fatigue neighboring pores could fuse forming larger objects in the processed images. A considerable increase of the amount of large objects in fatigued samples (Fig. 5), especially in plexiform ones, can indicate appearance of delaminations between collagen lamellae arising de novo or from fusion of preexisting shorter fissures. It was stated previously that damage severs collagen fibrils and the most frequently observed feature on the fracture surface in lamellar bone is inter-lamellar delamination [32–35].

The matter of discussion is, how much the shape and size of objects in microscopic image of demineralized samples reflects the shape and size of pores in native bone. Deposition of mineral in maturating bone tissue creates prestrains dependent on the local structure of collagen matrix [7]. Demineralization removes these prestrains and can cause small, anisotropic displacements of collagen fibrils and layers. On the other hand, dehydration being a part of staining procedure decreases molecular diameter of collagen [36] and can influence diameter of the fibrils, but did not create additional damage [37]. So, both demineralization and dehydration can influence size of collagen fibers, as well as the voids between them. However, there is no reason to presume that demineralization itself will create new voids within the organic matrix. So as, it can be presumed that all objects found in images of decalcified bone were present before demineralization, whether as a result of natural processes, or induced by fatigue pre-treatment. Structures of demineralized matrix examined in our study (Figs. 2a, 4) are similar to the both types of cortical structure in bovine bone with mineral in situ presented in the literature [2, 3]. One can assume that a kind of collapsing of the structures resulting from removal of mineral from the bone matrix did not influence significantly their spatial arrangement.

The next question addressed in the study was, how the fatigue induced changes in bone influence the ability of collagenous matrix to carry loads. Demineralized bone was extremely susceptible to compression and revealed its anisotropic nature. Extreme deformability of demineralized bone matrix is a consequence of the fact that it consists only of soft, compliant collagen fibers, basically without any possibility to transfer of loads between them, which results from removing of mineral phase. Crosslinks between and within fibers in bone tissue are not numerous and exist mainly at terminal parts of collagen subunits [38]. Very high failure strain in demineralized bone in compression (approximately 50 %) was reported also by Vashishth et al. [18]. However, in experiments on tensile behavior of demineralized bone ultimate strains were significantly lower [20, 22, 23, 25].

None of the axially loaded samples (L) failed within the applied range of deformation, and the level of stresses in L groups, was considerably lower than in T (transversely loaded) samples at the same strain (Fig. 6). The extremely high deformability in longitudinal direction may result from buckling of longitudinally aligned layers of soft collagen fibers in the absence of mineral.

A highly anisotropic nature of demineralized cortical bone was recently reported also in our other papers [27, 39] and in an detailed study by Novitskaya et al. [24]. Unfortunately, in the present study we were not able to analyze the degree of anisotropy in a quantitative way, because the ultimate failure of L samples was not achieved for deformation applied in the experiment.

It is difficult to compare mechanical parameters obtained in this study with values from other experiments on demineralized bone as mechanical behavior of bone depends on the method of loading [40, 41] and strain rate [12, 40]. In the experiment of Vashishth et al. [18] bone matrix was tested under compression but in a relaxation procedure. The other studies were conducted under tension [22, 23, 25]. The experiments were performed at strain rates different from ours and using some preconditioning procedures. However, strength at failure obtained in our experiment in a perpendicular direction is similar to those showed by others [23–25].

Another issue is a significant difference in strain at failure in the transverse direction between two histologically different types of samples obtained in this study. It is presumable that squeezing and shifting of soft and compliant collagen layers in an absence of large voids that can act as stress concentrators allows for a significantly larger deformation in the plexiform than in Haversian samples where both a fraction of large pores and the average pore size were considerably higher than in the plexiform samples.

With regard to impact of fatigue pretreatment on mechanical parameters, the only parameter that was significantly influenced was strain at failure in the transverse direction. The main contribution to that result was from a significant decrease of strain in A-M (plexiform) samples from fatigued bone. The decrease of strain in this group was accompanied by an increase of the average area of pores and more than threefold increase of the fraction of large pores.

An additional insight into the failure processes in collagenous bone matrix was obtained from analysis of acoustic emission (AE) from samples during compression. In material subjected to external loading a sudden redistribution of stress can cause local instabilities arising from heterogeneity of the sample resulting in local dynamic dislocation. The disturbed area can be a source of stress waves propagating in the form of acoustic emission. The amount of energy released by AE is related to the magnitude and velocity of the source events [42, 43]. Several attempts have been made to apply this method to study processes of bone fracture [27, 42–46]. In normal bone samples AE signals usually tended to initiate prior to the yield point and were continued during the non-linear region of bone deformation [44–46], while in demineralized bone AE was in the form of continuous, low energy emissions [44, 45]. AE is usually attributed to microcracks formation or propagation, but signals can be generated also as a result of friction or debonding between layers of the material [42]. So, in demineralized bone AE signal may result from microfractures as well as from a shifting and friction between collagen lamellae. In the present study, a continuous uniform emission of acoustic signals with low energy was accompanied by regular occurrences of high energy events. That pattern of AE distribution may result from both a shifting and friction between collagen lamellae and their gradual separation or debonding.

From the analysis of AE combined with the results of image analysis, it can be also concluded that AE is considerably related to the largest voids in the tested material. In P-L samples both AE and percentage fraction of large pores was high, both in control and in fatigued samples. The lowest level of AE was found in control A-M samples with the lowest fraction of large objects, but both AE and that fraction rose significantly in fatigued A-M group.

The question arises, why the same fatigue procedure applied to bone samples provoked different results in organic matrix from plexiform and from Haversian tissue. A possible answer is a presence of ductile matrix-osteon interfaces in haversian tissue. These cement lines composed from weakly bonded non-mature mineral decrease stiffness and strength of cortical tissue [6, 47] but on the other hand act as barriers absorbing cracks energy and protecting osteons from failure [9, 33]. It is presumable that in Haversian samples the main result of fatigue applied to bone was an increase of fissures between osteons without destroying their collagenous structure, so that their ability to absorb energy of deformation in demineralized samples was not disturbed. Plexiform bone has greater stiffness than Haversian one [15], but may lack the crack arresting properties, so the energy of fatigue could result in delamination of collagen layers and a consequent decrease in mechanical competence of demineralized matrix.

In summary, the study demonstrated that the effect of bone fatigue on collagenous matrix in cortical bone depended on the type of the bone structure. Even though fatigue procedure has induced structural changes in demineralized matrix in both types of tissue, the changes as well as the impact of fatigue on the response of samples to compression were more pronounced in the plexiform than in Haversian bone tissue. Demineralized matrix from both control and fatigued bone was anisotropic under compressive loads in both types of cortical structure.
